# Understanding of Hormonal Regulation in Rice Seed Germination

**DOI:** 10.3390/life12071021

**Published:** 2022-07-09

**Authors:** Diankai Gong, Fei He, Jingyan Liu, Cheng Zhang, Yanrong Wang, Shujun Tian, Chi Sun, Xue Zhang

**Affiliations:** 1 Liaoning Rice Research Institute, Shenyang 110115, China; gdkrice0709@126.com (D.G.); zhch024@163.com (C.Z.); wangyanrong518@163.com (Y.W.); tianshujunwy@163.com (S.T.); 15840417512@163.com (C.S.); 2 Tianjin Key Laboratory of Crop Genetics and Breeding, Tianjin Crop Research Institute, Tianjin Academy of Agricultural Sciences, Tianjin 300384, China; hefei1990@hotmail.com (F.H.); liujingyan0826@163.com (J.L.)

**Keywords:** seed germination, rice, abscisic acid, gibberellin, hormone signaling, transcription factors

## Abstract

Seed germination is a critical stage during the life cycle of plants. It is well known that germination is regulated by a series of internal and external factors, especially plant hormones. In *Arabidopsis*, many germination-related factors have been identified, while in rice, the important crop and monocot model species and the further molecular mechanisms and regulatory networks controlling germination still need to be elucidated. Hormonal signals, especially those of abscisic acid (ABA) and gibberellin (GA), play a dominant role in determining whether a seed germinates or not. The balance between the content and sensitivity of these two hormones is the key to the regulation of germination. In this review, we present the foundational knowledge of ABA and GA pathways obtained from germination research in *Arabidopsis*. Then, we highlight the current advances in the identification of the regulatory genes involved in ABA- or GA-mediated germination in rice. Furthermore, other plant hormones regulate seed germination, most likely by participating in the ABA or GA pathways. Finally, the results from some regulatory layers, including transcription factors, post-transcriptional regulations, and reactive oxygen species, are also discussed. This review aims to summarize our current understanding of the complex molecular networks involving the key roles of plant hormones in regulating the seed germination of rice.

## 1. Introduction

Crop seeds are the main source of food and fundamental materials in agricultural production. For improved production, the main agricultural goal is to obtain rapid and uniform germination and seedling emergence once seeds are sown, bringing up the point that germination is considered one of the most critical phases in the plant life cycle [1]. Rice (*Oryza sativa* L.) is one of the most principal foods in the world. Seed germination has a considerable impact on the yield and quality of rice production. Low seed germination and seedling uniformity lead to a significant reduction in rice production. Seed with rapid and uniform germination is called high-vigor seed, which is essential for vigorous seedling growth, tolerance to adversity, and high yield production. Meanwhile, the inappropriate loss of seed dormancy often results in pre-harvest sprouting, causing substantial losses in the yield and quality of rice [2]. This phenomenon is particularly serious during the production of hybrid rice in southern China due to the long-term rainy weather during harvest season. Aside from that, the emerging rice direct-seeding technology has received much attention because of its low input demand compared with conventional transplantation [3]. However, poor seed germination and seedling emergence in the field are the major constraints of direct-seeding systems. Thus, rice varieties with excellent germination ability (i.e., rapid and uniform germination and seedling emergence in the field context) are required.

Plentiful research and significant progress in understanding seed germination have been made in recent decades. By definition, germination commences with the absorption of water by the quiescent dry seed and is completed with the appearance of the radicle through the surrounding structures [4]. Following the physiological timeline of germination, numerous remarkable reviews have summarized the major physical and metabolic events that occur during germination, including imbibition, resumption of respiration, reserve mobilization, translation, transcription, cellular repair, and radicle extension [1,4,5,6,7]. The emerging area of seed research aims to identify the key regulators controlling germination. In recent years, the regulatory mechanism of germination has been extensively studied in *Arabidopsis* through molecular genetic studies. Many specific genes correlated with altered seed germination or dormancy have been identified, and the regulatory network, mainly consisting of hormone pathways and environmental factors, has been thoroughly proposed [2,8,9,10,11,12,13,14,15,16]. Unfortunately, the regulatory mechanism of rice, being a model monocotyledon plant and one of the most important crops, has not been explored as much as that in *Arabidopsis*.

The transition of seeds from a dormant state to germination is the result of the co-regulation of internal and external environmental factors. Environmental factors regulate endogenous components and thus affect the germination of seeds. Hormonal regulation plays a crucial role in this process by regulating the expression of related genes and signal transductions, which might be a highly conserved mechanism among seed plants [8]. ABA (abscisic acid) and GAs (gibberellins) are known to be the primary phytohormones that antagonistically regulate seed dormancy and germination. ABA positively regulates dormancy induction and maintenance, while GAs promote seed germination [17]. Changes in the balance of a seed’s ABA and GA levels and sensitivity influence the dormancy or germination state. That aside, nearly all other classes of plant hormones, including ET (ethylene), BRs (brassinosteroids), auxin, JA (jasmonic acid), SA (salicylic acid), CTKs (cytokinins), and SLs (strigolactones), regulate seed dormancy and germination, most likely by participating in the ABA or GA pathways [2,18]. Overall, hormonal regulation constitutes a central regulatory mechanism underlying the maintenance of seed dormancy and the onset of germination.

Recently, the regulation of rice germination by hormones has drawn much more attention, and several examples of innovative progress have been made. Rice seed germination possesses conserved features compared with *Arabidopsis* but also has distinct characteristics of its own. In this review, the established knowledge from *Arabidopsis* serves as a “backbone”, with the latest developments in rice research integrated. This review attempts to comprehensively illustrate the regulatory layers controlling the seed germination of rice. It will guide rice production and new variety breeding in agricultural practice.

## 2. ABA: The Core Factor in the Maintenance of Seed Dormancy and Inhibition of Seed Germination

### 2.1. ABA Metabolism Influences Rice Seed Dormancy and Germination

**Figure 1:**
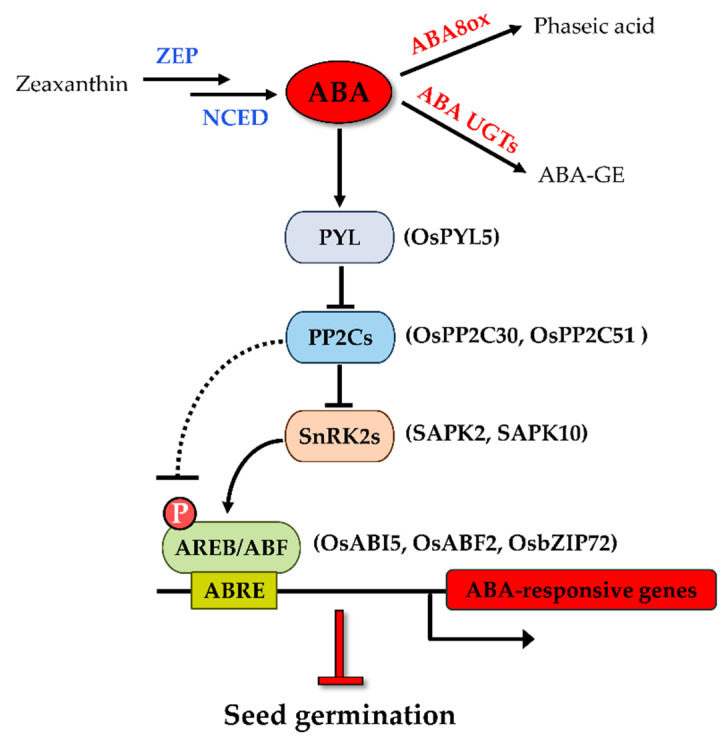
Key components of ABA metabolism and signaling pathway in rice seed germination.

The level of cellular ABA is determined by the dynamic balance of biosynthesis and catabolism of the hormone [19]. Zeaxanthin epoxidase (ZEP) catalyzes the conversion of zeaxanthin to violaxanthin in the upstream of the ABA biosynthetic pathway (Figure 1). In rice, a strong viviparous mutant impaired in ZEP named *Osaba1* has been isolated [20]. Nine-cis-epoxycarotenoid dioxygenase (NCED), cleaving the cis-isomers of violaxanthin and neoxanthin to xanthoxin, has been reported to be the rate-limiting step of ABA biosynthesis [21]. Changes in endogenous ABA levels are closely related to the expression of *NCED* transcripts. Seeds overexpressing *AtNCED6* had increased ABA levels during imbibition and were sufficient to prevent germination [22]. ABA catabolism largely relies on the hydroxylation of ABA at the 8′ position under the catalytic action of ABA 8′-hydroxylase (ABA8ox), which is encoded by *CYP707A* genes [19]. The most abundant ABA hydroxylated catabolite is phaseic acid (PA). A mutation of the *CYP707A2* genes in *Arabidopsis* showed an increase in ABA levels in dry and imbibed seeds and thus decreased germination potential [23]. ABA can also be inactivated by conjugation with glucose to form ABA glucosyl ester (ABA-GE) [19,24]. Several ABA glucosyl-transferases (UGTs) are capable of catalyzing this reaction. ABA-GE can be cleaved by β-glucosidases to form active ABA [19,24]. One of the MYB transcription factor members was recently reported to mediate the deconjugation of ABA-GE to ABA at a very early imbibition time and optimize seed germination in rice [25].

The expressions of the *NCED* and *ABA8ox* genes play significant roles in the control of the ABA level and therefore seed dormancy and germination. There are five members of the NCED gene family in rice. The expression pattern of the *OsNCED* transcripts was often used as a marker of the ABA level, despite their function during seed germination being far from investigative. The promoted germination or reduced dormancy phenotype was often accompanied by downregulation of all *OsNCEDs* but *OsNCED1* [26,27,28,29,30,31,32]. Similarly, it can be inferred that a reduction in the expression of the catabolic genes *OsABA8ox1*, *OsABA8ox2*, and *OsABA8ox3* inhibits seed germination [33,34].

ABA is necessary for seed maturation and the maintenance of dormancy. In *Arabidopsis*, there are two peaks of ABA accumulation that occur during the middle and late phases of seed maturation, of which the latter peak plays a key role in inducing and maintaining seed dormancy and thus preventing vivipary [2,35]. However, a study on rice implicated that the accumulation of ABA occurs at the earlier stage of seed development and then declines and remains low at the late stage [36]. The ABA level of a deep dormant cultivar was higher during the early and middle stages of seed development (10–20 DAP) than that observed in the low dormancy cultivars [36]. A similar result showed that the ABA accumulation peak appears at 10 DAP in hybrid rice seeds [37], suggesting the distinct mechanism of seed dormancy induction between rice and *Arabidopsis*. Aside from the induction of dormancy in developing seeds, ABA is involved in maintaining dormancy during imbibition [19]. Germination is determined by the decreased endogenous ABA level in the imbibing seed, which results from both the suppression of de novo synthesis and the activation of catabolism [35,38]. ABA Levels decline from imbibition onward, which is conserved in rice, as demonstrated by Liu et al. [36]. Furthermore, previous studies indicated that the reduction of the ABA level during rice imbibition is mainly related to the increased expression of *OsABA8ox* genes rather than the downregulation of biosynthetic *OsNCED* genes [33,39].

### 2.2. ABA Signaling Negatively Regulates Rice Seed Germination

In addition to ABA metabolism, the ABA signaling-dependent pathway also affects seed dormancy and germination. There were five ABA insensitive mutants of *Arabidopsis* isolated by the screen of ABA-resistant germination [40]. Subsequent research revealed that these mutants are impaired in genes participating in ABA signal transductions, and they are known as *ABI1* (*ABA Insensitive 1*), *ABI2*, *ABI3*, *ABI4,* and *ABI5*. During seed germination, mutants of these genes are insensitive to ABA (enhanced germination and seedling growth compared with the wild type) and exhibit a reduced seed dormancy phenotype. In the past decade, great efforts in the elucidation of the core ABA-signaling pathway have been made. ABA signaling consists of three main components: ABA receptor PYR/PYL/RCAR (hereafter referred to as PYL) proteins, protein phosphatase 2C (PP2C, the negative regulators), and SNF1-related protein kinase 2 (SnRK2, the positive regulators). The ABA signaling cascade is initiated from the binding of ABA to PYLs, leading to the form of a complex with PP2Cs, thereby suppressing PP2C-mediated dephosphorylation of SnRK2s [12,41]. The *ABI1* and *ABI2* genes mentioned above belong to group A of the PP2C family, which negatively regulates ABA responses in *Arabidopsis* seed [42,43]. The activated form of SnRK2s subsequently phosphorylates ABRE-binding protein/ABRE-binding factor (AREB/ABF) transcription factors (TFs), which in turn activates the transcription of ABA-responsive genes [44]. Among the downstream transcription factors, ABI5, a member of the bZIP type transcription factor family, plays a central role in activating the ABA response in seeds [24]. Aside from that, two other TFs, ABI3 (B3 domain TF) and ABI4 (AP2 domain TF), have been reported to function together with ABI5 to induce the ABA-responsive genes. Both TFs act upstream of ABI5, and they are positive regulators of the expression of ABI5 during seed germination [45,46].

ABA signaling and its role in regulating seed dormancy and germination have been intensively studied in *Arabidopsis* [24]. The established knowledge provided great support for better understanding the ABA regulatory role of seed germination in rice. Thirteen rice PYL orthologues were identified based on amino acid sequence analysis of the AtPYLs, and three members were functionally investigated [47,48]. Transgenic plants overexpressing *OsPYL3*, *OsPYL5*, and *OsPYL9* were found to be hypersensitive to ABA during rice seed germination, indicating they are positive regulators of ABA signaling in germination. ABA signaling is mediated by physical protein–protein interaction between the PYLs and clade A PP2Cs. It was found that OsPYL5 interacts with OsPP2C30, a homolog of AHG3 (ABA hypersensitive at germination 3), which is a PP2CA known to function specifically in the seed germination of *Arabidopsis* [49], in an ABA-dependent manner [47]. Other OsPP2CAs bind to OsPYLs in diverse fashions and with different intensities [48]. Among the PP2CA genes, *OsPP2C51* is predominantly expressed in seeds and positively regulates seed germination in rice [50]. It is worth noting that OsPYL5 is the target ABA receptor that interacts with OsPP2C51 [50], implying that OsPYL5 is the major PYL for transducing ABA signaling in seed germination. In rice, the downstream SnRKs are designated as Osmotic Stress/ABA-Activated Protein Kinases (SAPKs) [51]. It seems like SAPK2 may predominantly mediate ABA signaling relative to the other SAPKs because SAPK2 interacts physically with OsPP2C30 and OsPP2C51 [47,50], although this is not the ABA-activated protein kinase [51]. A recent study thoroughly investigated the role of SAPK10 in rice seed germination [52]. The SAPK10 overexpression lines showed delayed seed germination and hypersensitivity to ABA. Auto-phosphorylation of SAPK10 on Serine 177 is pivotal to its function, which enables it to activate the downstream TFs. bZIPs are typical AREB or ABF TFs that can be phosphorylated by SnRK2, and they constitute the core downstream component in ABA signaling. ABI5 is a seed-specific bZIP TF-activating ABA signal transduction, and it negatively regulates seed germination in *Arabidopsis* [53]. Several ABA-regulated ABI5 homologs have been identified in rice such as TRAB1 [54] and OsbZIP10 [55], and their functions regarding germination were well studied. The G-box motifs (containing the core ACGT motif and known as ABREs) bound by bZIP factors are highly enriched in the upstream regions of the ABI5-regulated genes [24]. OsABI5 (also known as OREB1 or OsbZIP10) could bind to a G-box element and transactivate downstream gene expression. A germination assay showed that *OsABI5* has a similar function to *AtABI5* in the ABA signal pathway [55]. Subsequent studies demonstrated that OsABI5 could be phosphorylated by SAPK2 [47,50] or deactivated by dephosphorylation of the OsPP2C51 detour at SAPK2 [50]. As a group A bZIP factor, OsABF2 (OsbZIP46) binds to ABREs, and the *Osabf2* mutant showed a significantly decreased sensitivity to high levels of ABA at the germination stage [56]. OsbZIP72 was found to interact with SAPK10 after a yeast-two-hybrid screen from rice seed [52]. The *bzip72* mutant showed no change in germination as well as its closest homolog *trab1/osbzip66* mutant did, while its overexpression lines showed enhanced sensitivity to ABA, suggesting OsbZIP72 negatively regulates seed germination. The above research indicates that AREBs, ABFs, and ABI5 are the major positive downstream regulators of ABA signaling during rice seed germination. That aside, as mentioned above, ABI3 and ABI4 are also critical to seed germination because they are positive regulators of ABI5. Their physiological functions in rice will be discussed in the following section. Collectively, these observations provide a core ABA signaling pathway in rice seed germination, consisting of PYL-PP2Cs-SAPKs-bZIPs (Figure 1). Any modification of components in this ABA response pathway can result in changes in the germination potential.

## 3. GA Promotes Rice Seed Germination by Acting Antagonistically to ABA

### 3.1. GA Metabolism Influences Rice Seed Germination

**Figure 2:**
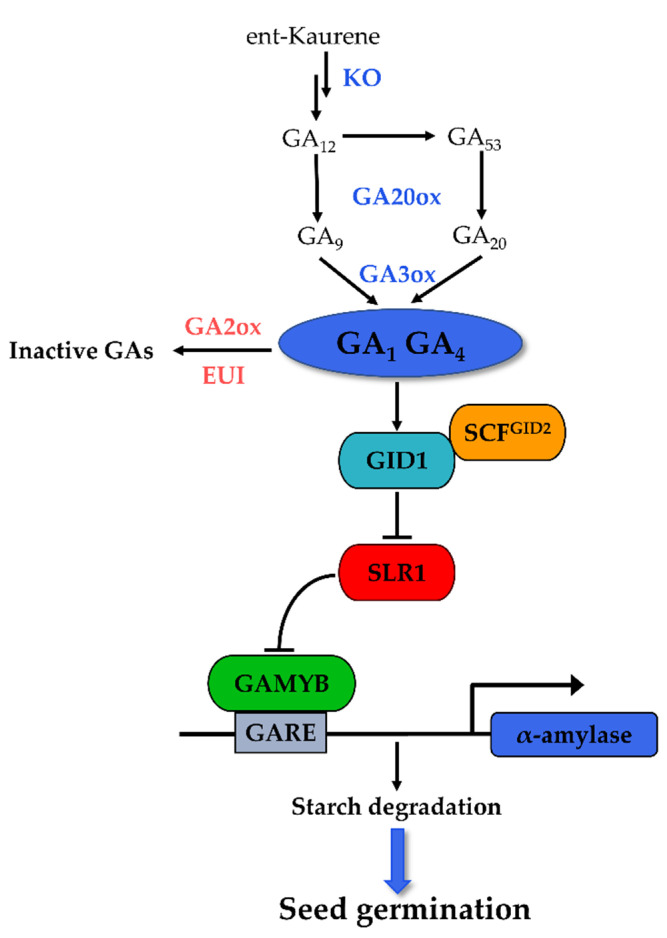
Key components of GA metabolism and signaling pathway in rice seed germination.

GA plays an antagonistic role to ABA in the regulation of seed germination. The positive regulation of GA in seed germination is determined by the balance between its biosynthesis and inactivation [57]. Bioactive GA is formed in terminal reactions mainly catalyzed by GA 20-oxidase (GA20ox) and GA 3-oxidase (GA3ox), while its inactivation is controlled primarily by GA 2-oxidase (GA2ox) (Figure 2). The endogenous bioactive GA contents and seed germination are correlated with the expression levels of these enzyme-encoding genes in rice. The expression of *OsGA3ox2* in an embryo is important for α-amylase induction during rice seed germination [58], while that of GA-inactivating genes (*GA2ox*) is repressed during the imbibition of non-dormant rice [39]. The *Seed Dormancy1-2* (*qSD1-2*) locus contains a GA synthesis gene *OsGA20ox2*, and the loss of function mutation in *OsGA20ox2* leads to reduced seed GA levels and enhanced dormancy [59]. Likewise, *OsGA2ox3* was identified as a candidate gene for controlling seed germination [60]. In a *germination-defactive1* (*gd1*) mutant, the expressions of GA biosynthesis genes *OsGA20ox1*, *OsGA20ox2*, and *OsGA3ox2* were suppressed, while that of the inactivation gene *OsGA2ox3* was dramatically upregulated, resulting in decreased endogenous GA_4_ content and the inhibition of seed germination [61]. Another key enzyme, ent-kaurene oxidase (KO), is involved in the early steps of GA biosynthesis, converting ent-kaurene to ent-kaurenoic acid [57]. The *OsKO1* gene might function mainly at the seed germination and young seedling stages. The defect in this gene leads to a decreasing GA content and shows both delayed germination and the semi-dwarfism phenotype [62]. In addition, OsLOL1 promoted seed germination through the upregulation of *OsKO2* [63], suggesting the important role of *OsKOs* during rice germination.

### 3.2. GA Signaling in the Regulation of Rice Seed Germination

The central GA signaling components include the soluble GA receptor GIBBERELLIN INSENSITIVE DWARF1 (GID1, a positive regulator), the DELLA proteins (negative regulator), and the F-box proteins GID2 (positive regulator, SLEEPY 1 (SLY1) in *Arabidopsis*) [64]. Bioactive GA is perceived by its receptor GID1, leading to the formation of the GA-GID1-DELLA complex [65]. This complex associates with the F-box protein, the central component of SCF^GID2^ E3 ubiquitin ligase, resulting in DELLA degradation via the ubiquitin-26S proteasome pathway [64]. The degradation of DELLA proteins activates the downstream GA response TF, typically the GAMYB, and thus mediates the GA signaling effects [66].

As positive regulators in GA signaling, the *GID1* and *GID2* genes are supposed to promote seed germination, just as found in the observations of *Arabidopsis* [67,68]. However, the mutation in *GID1* and *GID2* for the rice does not appear to affect seed germination, although their mutations repress the α-amylase activity, and the expression of *GID2* is not associated with the seed germination phenotype [35]. These results suggest that the response to GA signaling in rice seeds may be independent of the *GID1* and *GID2* levels. The homolog of DELLA proteins in rice was identified as SLENDER RICE1 (SLR1) [69]. There is no direct evidence about the role of SLR1 in germination, but a study on wheat showed that inhibition of the germination of non-dormant seeds is associated with increased expression of *RHT1* (DELLA of wheat) [70]. Therefore, it is reasonable that the degradation of SLR1 is essential to rice seed germination.

The molecular mechanisms of downstream transcriptional regulation of GA-responsive genes have been extensively studied, and the response of α-amylase genes in aleurone cells to GA is considered the best-characterized GA response [64]. Many pivotal *cis-*acting elements and TFs involved in this response were proposed, of which GAMYB plays the central role. GAMYB is a classic MYB TF. In response to a GA signal, it binds to the GA-responsive elements (GAREs) of α-amylase, positively regulating the expression of the gene in the aleurone of rice seeds [71]. Another TF such as DNA binding with one finger (DOF) protein was shown to interact synergistically with GAMYB as a functional unit in the transcription regulation of the GA-responsive *RAmy1A* gene in rice seed [71], while two WRKY domain TFs, OsWRKY51 and OsWRKY71, worked as a transcriptional repressor of GA signaling by suppression of the OsGAMYB-activated *Amy32b* expression [72,73,74]. In summary, GA signal cascades during rice seed germination include the following steps. SLR1 binds to GAMYB in the absence of GA, resulting in the inhibition of α-amylase. When GA is present, the formation of a GA-GID1-SLR1 complex is promoted, which facilitates the SCF^GID2^-mediated degradation of SLR1 through the ubiquitin-proteasome pathway. The released GAMYB from SLR1 then binds to the GARE element in the promoter of α-amylase and activates their expression, consequently leading to the hydrolysis of starchy endosperm reserves (Figure 2).

## 4. ABA and GA Balance Controls Seed Germination in Rice

ABA or GA does not act solely in controlling seed germination; the dynamic balance between ABA and GA is more critical in this process. What we are concerned about is the crosstalk between these two hormones; that is, ABA and GA reciprocally regulate the metabolism and signal transduction of each other [75].

Dormancy release or germination is often accompanied by decreased ABA levels and increased GA levels, which are contributed by the altered expression patterns of synthetic and catabolic genes. A study indicated that the embryo-imposed dormancy is mainly determined by the ABA/GA ratio at the developmental stage of rice seed. Rice cultivars with a deep dormant phenotype have high ABA/GA ratios and relatively high transcript levels for the key genes in the ABA and GA metabolism pathways [36]. It is well known that ABA suppresses the synthesis of GA and promotes its deactivation, both of which result in a lower GA content in the seeds and lower completion of germination of *Arabidopsis*. On the contrary, GA also negatively regulates ABA biosynthesis. The expression of ABA biosynthetic genes is increased in GA-deficient mutants, resulting in the accumulation of higher ABA levels [12]. A similar observation is also available for rice. In the *OsKO1* and *OsKO2* (encoding GA synthetic enzyme) mutants, most of the GA biosynthetic genes and ABA catabolic genes were upregulated, whereas ABA biosynthesis genes were downregulated [62]. Aside from that, trans-genetic lines or mutants generated in rice showing altered germination phenotypes are usually accompanied by a change in expression patterns of synthetic and catabolic genes in the ABA and GA metabolism [76,77]. All results indicate the balance of the ABA and GA levels being crucial to rice seed germination.

The ABA/GA balance is also controlled by mutually antagonistic regulation at the signal transduction layer. Some components of the ABA signal cascade were also found to be regulated by GA. Rice ABA receptor OsPYLs could be degraded by Tiller Enhancer (TE), an activator of the APC/C^TE^ E3 ubiquitin ligase complex [78]. The presence of ABA inhibits APC/C^TE^ activity by phosphorylating TE through activating SnRK2s, while GA can repress the activity of SnRK2s and may promote the degradation of OsPYLs. Thus, the loss-of-function *te* mutant displays decreased seed sensitivity to ABA and enhanced germination. This work proposed a novel signaling hub mediating the antagonistic action of ABA and GA in plants. Emerging evidence indicates that AP2 domain TFs, one of the important TFs in response to ABA, play key roles in ABA and GA antagonism [79]. In *Arabidopsis*, ABI4 promotes ABA accumulation through repressing expression of the ABA-inactivating genes *CYP707A1* and *CYP707A2* but negatively mediates GA biosynthesis during the seed germination processes [80]. Though no direct target of GA metabolism genes by ABI4 has been detected, ABI4 of sorghum can directly bind to the promoter of SbGA2ox3 to activate its expression, thereby causing a reduction in GA and maintaining seed dormancy [81]. As with ABI4, a rice AP2 domain TF, OsAP2-39, regulates germination by modulating the balance between the ABA and GA levels [76]. OsAP2-39 upregulates transcription of the ABA biosynthesis gene *OsNCED1* and also enhances expression of the GA-inactivating gene *OsEUI* (elongated uppermost internode). Furthermore, transcription of the *ABA responsive AP2-like* (*ARAG1*) gene was enhanced in a GA-deficient mutant of rice [62]. Taken together, further research on AP2 domain TFs is needed to improve our understanding of the ABA/GA crosstalk, especially in seed germination.

On the other hand, regulators in the GA signaling pathway also influence the regulation of ABA biosynthesis. In addition to the negative role in GA signaling, DELLA proteins act as regulators of GA and ABA crosstalk in germination. DELLA protein RGL2 is a major GA signaling repressor in the germination of *Arabidopsis*. Inhibition of seed germination by RGL2 is probably achieved by enhanced ABA synthesis and ABI5 activity [82]. When the GA level is low, RGL2 stimulates the expression of *XERICO*, a gene encoding a RING-H2 zinc finger factor promoting ABA accumulation [83]. In turn, increased endogenous ABA synthesis is necessary to elevate ABI5 at both the RNA and protein levels. The increased ABI5 protein is ultimately responsible for preventing seed germination [82]. Consistently, overexpression of *XERICO* in rice exhibited hypersensitivity to exogenous ABA during seed germination [84]. The transgenic lines exhibited a significant increase in the endogenous ABA contents and expression levels of *OsNCED* and *OsABI5*. Aside from that, as several other studies have demonstrated the regulatory role of DELLA proteins and ABI5 interaction [85,86], a detailed understanding awaits further study for the balance of ABA and GA in rice germination.

## 5. Other Hormones Involved in the Regulation of Rice Seed Germination

**Figure 3:**
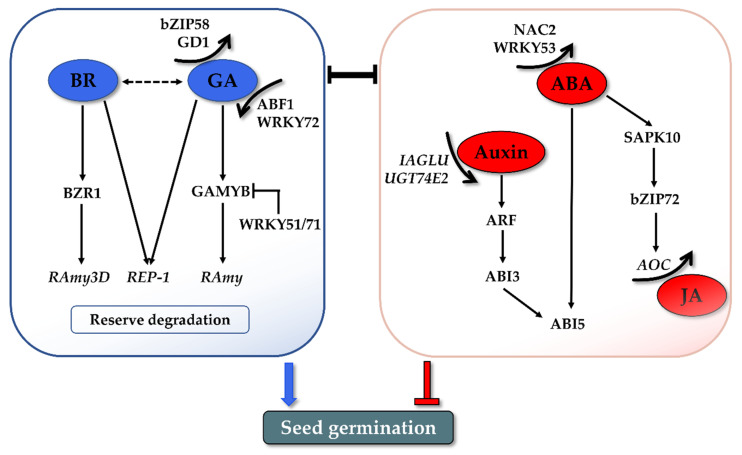
Preliminary network of hormonal regulation in rice seed germination. GA and BR synergistically promote seed germination through reserve mobilization. Auxin and JA inhibit germination through the crosstalk with ABA. Numerous TFs play crucial roles in connecting upstream hormonal signals and regulating the downstream gene expressions. The interaction of various hormones and TFs influences the balance of ABA and GA, which is the core determinant of seed germination.

BR was found to promote seed germination. In *Arabidopsis*, BR can rescue the germination phenotype of severe GA-deficient and GA-insensitive mutants [87]. In addition, germination of both the BR-biosynthetic and BR-insensitive mutants is more strongly inhibited by ABA, suggesting the role of BR in reducing ABA sensitivity during germination [87]. A further study demonstrated that Brassinosteroid Insensitive 2 (BIN2), the key negative regulator in the BR signaling pathway, could phosphorylate and stabilize the ABI5 protein to antagonize the inhibitory roles of ABA on seed germination [88]. All these observations indicated that the positive role of BR is integrated with the balance of ABA/GA in seed germination. The promotion role of BR has also been verified in rice germination. The germination rate was reduced in the presence of BRZ, a BR biosynthesis inhibitor, to mimic BR-deficient germination conditions [89]. A mutation of notched belly grain4 (*nbg4*), which is an allele of *Dwarf 11* encoding cytochrome P450 (CYP724B1), which is involved in BR biosynthesis, also displayed retarded germination [90]. A recent study further investigated the possible mechanism by which GA and BR coordinate seed germination through storage protein mobilization (Figure 3). BR and GA can promote the expression of cysteine proteinase (REP-1) during germination, leading to degradation of the stored glutelin in the endosperm to produce more amino acids, thus facilitating the growth of an embryo [91]. In addition, a novel regulatory module was found independent from the GA pathway [92]. A key downstream TF of the BR signaling cascade, brassinazole-resistant 1 (BZR1), mediates rice seed germination by binding to the *α-Amylase 3D* (*RAmy3D*) promoter and activates α-amylase expression and activity. This BZR1-RAmy3D transcriptional module then regulates the degradation of starch in the endosperm, thereby promoting seed germination [92]. More systemic research is still needed to illustrate how BR regulates rice seed germination.

Auxin was reported to be involved in maintaining seed dormancy and inhibiting seed germination. Earlier studies showed that exogenous application of the auxin indole-3-acetic acid (IAA) delayed seed germination in wheat and soybean [93,94]. No such direct evidence is available in rice, but studies indicated that the changed endogenous IAA levels influence rice seed germination. Conjugate modification is one of the important processes that tightly controls the active auxin levels, by which the auxin glycosyltransferases catalyze the transfer of activated sugars to auxin [95]. IAA glycosyltransferase (*IAGLU*) was recently identified in rice and involved in catalyzing IAA glycosylation [96]. The higher levels of free IAA were identified in the germinating seeds of *osiaglu* mutants with lower germination speeds compared with the wild type. Similarly, the overexpression of UDP-glucosyltransferase 74E2 (*UGT74E2*), which transfers glucose to indole-3-butyric acid (IBA), could enhance seed germination in rice, which is partially attributed to the lower levels of free IBA [97]. The inhibitory effects of auxin brought out the question of what the mechanism by which auxin controls seed germination is. Emerging data showed that auxin regulates seed dormancy and germination through crosstalk with ABA. The auxin-responsive transcription factors ARF10 and ARF16 act upstream of ABI3 by indirectly promoting its transcription and consequently maintaining seed dormancy and repressing germination [98]. This crosstalk between the auxin and ABA signaling pathways is highly consistent with the observations in the recent studies on rice. Disruption of *OsIAGLU* elevated both the IAA and ABA contents and continuously induced the expression of *OsABIs* [96]. Accordingly, both the IBA and ABA levels declined in *UGT74E2*-overexpressing plants, and their signaling pathways were also affected, including the downregulation of *ARF*, *ABI3*, and *ABI5* (Figure 3) [97].

JA has been proposed to inhibit rice seed germination at the metabolism and signaling level. The application of Methyl Jasmonate (MeJA) effectively inhibited the germination of rice seeds [99]. In the transgenic rice plants over-accumulating JA, germination was significantly retarded compared with the wild-type [100]. That aside, *coi1* mutants of rice, a key component of a JA receptor, exhibited faster germination phenotypes, indicating its negative regulatory role in germination [101]. The inhibitory effect on the germination of JA is usually attributed to its working synergistically with ABA. As a key metabolic intermediate during JA biosynthesis, OPDA acts along with ABA to inhibit seed germination in *Arabidopsis* [102]. Jasmonate-ZIM domain (JAZ) protein is the repressor of JA signaling. JAZ was revealed to negatively modulate ABA-inhibited seed germination through interaction with ABI5 in bread wheat and *Arabidopsis* [103]. JAZ3 in bread wheat interacts with ABI5 and represses its transcriptional activation activity, thus resulting in repression of the ABA signal, thereby promoting seed germination [103]. The synergistic effect of ABA and JA in germination inhibition has been recently validated in rice. As described in Section 2.2, the interaction of SAPK10 and bZIP72 constitutes a critical pathway for ABA signaling in rice seed germination [52]. Interestingly, one of the downstream targets activated by bZIP72 is allene oxide cyclase (*AOC*), which is a JA biosynthesis step-limiting gene. The germination assays in this work also indicated that ABA-inhibited germination is partially based on the activated JA level. In summary, the most compelling evidence of the ABA- and JA-participating pathway in rice seed germination is as follows (Figure 3): exogenous ABA confers auto-phosphorylation of SAPK10, which activates and enhances bZIP72 stability as well as the DNA’s binding ability to the G-box *cis*-element of the *AOC* promoter, and the elevated *AOC* transcription finally increased the endogenous JA level to synergistically inhibit seed germination [52].

## 6. Transcriptional Regulation of Hormonal Signaling during Rice Seed Germination

Transcription factors play key roles in connecting upstream signals and downstream transcriptional networks, which contribute to the adaptation of plants to the changing environment. To date, several classes of TFs have been demonstrated to be essential for the ABA- or GA-regulated gene expression during rice seed germination: B3, AP2, bZIP, WRKY, and NAC (Table 1).

### 6.1. TFs Participate in ABA-Regulated Germination

As the ortholog of maize Viviparous1 (VP1) [104], ABI3 represents a classic B3 domain TF member in response to ABA-inhibited germination in *Arabidopsis* [105]. The positive role of ABI3/VP1 in ABA signaling is achieved by interacting with AREB/ABF/ABI5, which directly binds to ABREs in ABA-regulated genes. ABI3 acts upstream of ABI5 and is essential for *ABI5* expression to arrest the growth of germinating embryos [45]. The conserved evidence has also been addressed in the OsVP1 of rice [54]. *Oryza sativa* Delayed Seed Germination 1 (*OsDSG1)* encodes a RING finger E3 ligase that negatively regulates ABI3 [106]. The *osdsg1* mutant showed a delayed germination phenotype, together with significantly increased transcript levels of ABA-signaling genes such as *OsABI3* and *OsABI5* [106]. Thus, if the degradation of OsABI3 mediated by OsDSG1 is possibly needed in germinating rice seeds, then the ABA signal cascade is blocked via the OsABI3–OsABI5 pathway, and finally the ABA-inhibited genes (such as those genes encoding hydrolytic enzymes) reactivate, resulting in the promotion of germination.

As an AP2 domain-containing TF, ABI4 positively regulates the primary seed dormancy in *Arabidopsis* by directly binding to the promoters of *CYP707A1* and *CYP707A2*, the ABA catabolism enzyme-encoding genes, subsequently promoting ABA accumulation [80]. The specific role of the ABI4 homolog in rice seed germination has not been detected thus far. Within the AP2 domain family, ABI4 is most closely related to the Drought Response Element Binding (DREB) subfamily [24]. A DREB-like gene, ABA responsive AP2-like gene (*ARAG1*), was demonstrated to play a role in rice seed germination [107]. Knockdown of *ARAG1* conferred hypersensitivity to ABA inhibition during seed germination and seedling growth, suggesting its negative regulation in the ABA signaling pathway [107]. Furthermore, a transcriptomic study highlighted that the AP2 domain containing TFs, especially some *OsDREBs* enriched in the initial imbibition of rice seed germination [108]. Since AP2 domain TFs were considered to play key roles in seed dormancy regulation [2], further information is needed about the regulation role of the AP2 family in rice seed dormancy and germination.

The ABA signaling pathway mainly relies on the bZIP transcription factors. Among the large bZIP family members, the AREB/ABF/ABI5 subfamily binds to the ABREs, and OsABI5 (OsbZIP10) plays a key role in suppressing ABA-mediated seed germination (Section 2.2). In addition, several other bZIP members are also involved in the ABA-mediated regulation of seed dormancy and germination in rice. MOTHER OF FT AND TFL 2 (OsMFT2) was found to be a negative regulator of rice seed germination [109]. It delayed germination via interacting with three bZIPs (OsbZIP23, 66, and 72) and enhancing their binding to ABREs of *Rab16A*, a typical ABA-responsive gene. Notably, *OsbZIP23* overexpression could restore the PHS phenotype of *osmft2* knockout lines [109]. OsbZIP75 directly binds to the promoter of *DELAY OF GERMINATION 1* (*DOG1*, a major seed dormancy controlling gene) and promotes the accumulation of OsDOG1L-3 [110]. High expression of OsDOG1L-3 then induces the expression of ABA synthesis genes and increased endogenous ABA contents, leading to enhancing the ABA signaling, and finally, seed germination is inhibited [110]. Generally, OsbZIPs negatively regulate rice germination, but a recent study demonstrated that OsbZIP09 positively promotes seed germination [111]. Further evidence indicated that OsbZIP09 regulates seed germination via interacting with ABA signaling to coordinate the expression of common downstream target genes (i.e., by suppressing the expression of *OsLEAs* (negative regulators of germination) and enhancing the expression of *OsLOX2* (a positive regulator of germination)) [111].

WRKY TFs are key regulators of many plant processes, including seed dormancy and seed germination [112]. The involvement of WRKY TFs in seed germination or dormancy is determined by their response to ABA. As ABA-inducible genes, many WRKY TFs are involved in the suppression of germination by ABA. For example, seeds of the *AtWRKY2* knockout mutant showed an increased delay in germination in the presence of ABA, and the ABA-induced WRKY2 accumulation during germination requires the participation of ABI3 and ABI5 [113]. In rice, OsWRKY72 and OsWRKY77 enhanced transcription from the promoter of the ABA-responsive gene HVA22 under ABA treatment [114]. OsWRKY53 directly binds to the promoters of *OsABA8ox1* and *OsABA8ox2* (ABA catabolic genes) to repress their expression, leading to the accumulation of ABA and furthering the inhibition of seed germination [115]. However, WRKY TFs have functional differences in regulating seed germination, because several WRKY proteins were found to promote seed germination by negatively modulating ABA-responsive genes [116]. Likewise, the knockout and RNAi lines of *OsWRKY29* had enhanced seed dormancy, whereas its overexpression lines showed significantly increased germination [29]. Further assays showed that OsWRKY29 could bind to the promoters of *OsABF1* (ABI5-type TF) and *OsVP1* (ABI3-type TF) to inhibit their expression, thus resulting in a weakened ABA response and promoting seed germination [29].

NAC (NAM-ATAFCUC) TFs involve in various events during plant development [117], while their effects on seed germination in rice remain largely unknown. A recent study reported that a rice NAC family member, OsNAC2, negatively regulates seed germination [30]. Despite the binding ability to the promoter of ethylene biosynthetic genes, OsNAC2 probably inhibits germination through the ABA pathway instead of the ethylene and GA pathways. Ethylene is generally recognized to promote seed germination with an antagonistic function to ABA [2]. The results from OsNAC2 research indicate that ABA may play a more advantageous role in the regulation of seed germination in rice. Overall, further investigation is warranted to depict the interactions between ethylene and the upstream transcriptional regulation of NACs during seed germination.

### 6.2. TFs Participate in GA-Regulated Germination

In addition to ABI3, the ABI3/VP1-related subfamily of B3 domain TFs includes a family of VP1/ABI3-like (VAL) factors and two members of the *leafy cotyledon* class of regulators that control embryo maturation: Leafy Cotyledon1 (LEC2) and FUSCA3 (FUS3) [24]. These members play a central role in controlling the developmental regulation from embryogenesis to germination. LEC2, FUS3, and ABI3 serve as activators of seed development [118] while VALs repress it [119]. The rice mutant *germination-defective1* (*gd1*) is defective in seed germination and seedling development [61]. *GD1* encodes a B3 domain-containing TF with high similarity to *VALs* in *Arabidopsis*. In *gd1*, the inhibited germination is due to the decreased endogenous GA_4_ level [61]. These data indicate that the VAL subfamily participates directly in regulating GA homeostasis and further regulates rice seed germination and seedling development.

The G-box *cis*-element exists not only in ABA-inducible genes but also in the promoter of many genes in response to developmental and environmental signals, including GA biosynthesis gene *OsKO2*. OsbZIP58 binds the G-box *cis*-element of the *OsKO2* promoter and activates its expression, which promotes GA biosynthesis and seed germination [63]. Another piece of evidence of bZIP involvement in GA regulating germination has also been proposed recently. OsABF1, a member of the bZIP TFs, participates in many abiotic stress responses, including salt stress and drought stress, in the ABA-dependent manner [120,121]. However, OsABF1 also functions as a key repressor of GA synthesis in seed germination [122]. Overexpression of *OsABF1* showed a typical GA-deficient phenotype with semi-dwarf and retarded seed germination. Further, OsABF1 directly binds to the G-box in the promoter of *semi-dwarf 1* (*SD1,* which encodes GA20ox2 in GA biosynthesis) and suppresses its transcription, thus reducing the endogenous GA level in the seeds [122]. Regarding the roles in ABA signaling and GA biosynthesis suppression, the ABF subfamily of bZIPs is possibly the key hub in mediating the antagonism of GA and ABA.

WRKY genes were suggested to regulate the ABA and GA signaling crosstalk in seed germination. OsWRKY71 acts as a transcriptional repressor of the GA signaling by blocking the activation of the *Amy32b* promoter by OsGAMYB [72]. Further studies indicate that OsWRKY71 works with another OsWRKY51 to mediate crosstalk between the GA and ABA. The ABA-inducible and GA-repressible OsWRKY51 and OsWRKY71 TFs form a heterotetramer that binds the *Amy32b* promoter and prevents the activation of the promoter by OsGAMYB [73]. Moreover, in the mutant of *Ideal Plant Architecture 1* (*IPA1*) plants, seed germination and early seedling growth were retarded, together with the significantly upregulated expression of OsWRKY51 and OsWRKY71. IPA1 was further found to directly bind to and activate the expression of OsWRKY51 and OsWRKY71, which would interfere with the binding affinity of GA-induced OsGAMYB to inhibit the expression of a-amylase genes [74]. Aside from that, OsWRKY72 was recently identified as a negative regulator in rice germination through the “LRK1-OsKO2”; pathway in the aleurone layer [123]. WRKY72 directly targets LRK1 (a leucine-rich repeat receptor-like kinase) and activates its transcription, suppressing the ent-kaurene oxidase OsKO2 and thereby resulting in a reduced endogenous GA level and inhibited germination.

**Table 1:** Transcription factors involved in hormonal signaling during rice seed germination.

TF Class	Gene	Function Description	Role in Germination	Reference
bZIP	*ABI5/OsbZIP10*	Positively activates expression of ABA-responsive genes in seeds	Negative	[55]
	*ABF2/OsbZIP46*	Positively regulates ABA signaling, and germination is less sensitive to ABA in mutant	Negative	[56]
	*OsbZIP72*	Phosphorylated by SAPK10 and activates AOC transcription	Negative	[52]
	*TRAB1/OsbZIP66*	Interacts with VP1 and functions redundantly with *OsbZIP72* in ABA signaling	Negative	[52,54]
	*OsbZIP75*	Interacts with *DOG1* to increase endogenous ABA contents and ABA signaling	Negative	[110]
	*OsbZIP09*	Interacts with ABA signaling to coordinate the expression of common downstream target genes	Positive	[111]
	*OsbZIP58*	Activates *OsKO2* expression to promote GA biosynthesis	Positive	[63]
	*ABF1*	Suppresses the transcription of *SD1*, thus reducing the endogenous GA level	Negative	[122]
B3	*ABI3*	Acts upstream of ABI5 to positively regulate ABA signaling	Negative	[54,106]
	*GD1*	Similar to VALs in *Arabidopsis*, and mutant inhibits germination by decreasing endogenous GA_4_ level	Positive	[61]
AP2	*OsAP2-39*	Promotes the transcription of *OsNCED1* and *OsEUI*, thus influencing balance of ABA and GA	Negative	[76]
	*ARAG1*	Germination is hypersensitive to ABA in mutant	Positive	[107]
	*OsDREBs*	Enrich in the initial imbibition of seed germination	Not mentioned	[108]
WRKY	*OsWRKY72*	Enhances transcription of ABA-responsive gene and also suppresses *OsKO2* expression to reduce endogenous GA level	Negative	[114,123]
	*OsWRKY53*	Represses the expression of *OsABA8ox1* and *OsABA8ox2* to promote ABA accumulation	Negative	[115]
	*OsWRKY29*	Inhibits the expression of *OsABF1* and *OsVP1*	Positive	[29]
	*OsWRKY71*	Interferes with OsGAMYB to block GA signaling	Negative	[72,73,74]
	*OsWRKY51*	Functions in blocking GA signaling with OsWRKY71	Negative	[73,74]
NAC	*OsNAC2*	Overexpression line delays germination by modulating the expression of *OsNCED3*, *OsZEP1*, and *OsABA8ox1*	Negative	[30]

## 7. Post-Transcriptional Regulation of Seed Germination in Rice

Hormones mainly act as integrators between environmental cues and molecular signals for the transcriptional regulation of gene expression in seed germination. While many other layers of gene expression exist, these include mRNA splicing, epigenetic processing, translation, and degradation [16]. Alternative splicing of mRNA generates multiple transcripts from a single gene and leads to different mRNA variants. The unusual processing patterns in the *OsVP1* gene alter preharvest sprouting among rice varieties has been shown [124], with similar observations available in peas and wheat [125,126]. Epigenetic regulation involves histone protein modifications, represented by histone acetylation [127]. Deacetylation of histone is catalyzed by histone deacetylases (HDACs, which remove acetyl groups from histones), which is correlated with transcriptional repression [128]. The overexpression of *HDA705*, a member of HDACs in rice, delayed seed germination with downregulated expression of GA biosynthetic genes and the upregulation of ABA biosynthetic genes [129]. However, it is still unclear whether HDACs repress their expression through histone deacetylation. Protein ubiquitination is an important protein modification that negatively affects the stability of proteins. Ubiquitination of ABI3 plays a key role in germination [106]. Changes in ubiquitination were detected in 1171 proteins during rice seed germination, which especially occurred within the first 12 h [130].

## 8. Regulation of Rice Seed Germination by Reactive Oxygen Species

Reactive oxygen species (ROS) consist of the superoxide anion radical (O_2_^−^), hydrogen peroxide (H_2_O_2_), and the hydroxyl radical (•OH) [131]. Although ROS have long been considered toxic molecules by which ROS accumulation imposes oxidative stress on seed viability during desiccation or deterioration, increasing evidence shows that ROS function as signaling molecules to promote seed germination [131,132]. H_2_O_2_ is reported to mediate the regulation of ABA catabolism and GA biosynthesis during germination in *Arabidopsis* [133]. Links among ABA, ROS, and GA provide a possible explanation for the antagonism between ABA and GA in controlling seed germination [134]. The reduced ROS production by ABA in imbibed rice seeds also led to an inhibition of ascorbic acid (ASC) production. As a substrate in GA biosynthesis, insufficient ASC levels suppressed GA accumulation, which was indicated by the inhibited expression of GA biosynthesis genes [134]. These data suggest ROS and ASC are involved in the ABA-induced inhibition of GA biosynthesis. Furthermore, the ABA and H_2_O_2_ interaction during seed germination was investigated [135]. In the transgenic lines showing a delayed germination phenotype, the endogenous ABA levels were significantly higher, and the H_2_O_2_ levels significantly lower. Aside from that, exogenous H_2_O_2_ treatment can mostly recover the inhibited germination by ABA [135]. Moreover, it has recently been proposed that the improved seed vigor of *OsCDP3.10* might be through enhancing H_2_O_2_ accumulation in early germination [136]. All of these studies confirmed that seed germination is enhanced through ABA catabolism and H_2_O_2_ production. PER1 is a seed-specific peroxiredoxin antioxidant for scavenging ROS [137]. *PER1A* in rice positively regulates seed vigor under the control of the ABA signaling pathway [138]. Despite these reports, the molecular bases underlying ROS-mediated germination in rice seeds are still elusive.

## 9. Conclusions and Future Directions

The complex molecular networks that underlie successful seed germination involve the integration of environmental cues and hormonal signals. Utilizing forward and reverse genetic approaches in *Arabidopsis*, considerable progress has been made in the dissection of molecular mechanisms underlying the hormonal regulation in seed dormancy and germination. ABA is considered the main factor influencing seed dormancy and inhibition of germination, while GA acts as an ABA antagonist, promoting the germination of rice seeds. The metabolic and signaling pathways along which ABA and GA act are determinants, as well as the effect of the balance of ABA and GA on the germination of rice seeds. Some other hormones (brassinosteroids, auxins, and jasmonic acid) contribute to the regulation of rice seed germination, most likely by participating in the ABA and GA pathways. Although several key factors that regulate this important event have been identified in rice, a systematic network of hormone-regulated germination is still lacking, and many important questions remain to be answered.

The research on rice has presented the specific role of plant hormones in seed germination, including ABA, GA, BR, auxin, and JA. However, the involvement of other hormones, such as ET, SA, CTKs, or SLs, is still largely unknown. It will be interesting to explore their interactions with the ABA or GA pathways during germination in rice or even whether they can regulate germination independently like BR does [92]. Genetic studies screening for abnormal responses to these hormones can be employed based on their physiological phenotypes. Aside from that, many key components have been discovered to be involved in metabolism and the signaling pathways for these hormones in *Arabidopsis*. Functional characterization of their homologs in rice would greatly extend the complex molecular networks of hormones controlling seed germination. The emerging discoveries in *Arabidopsis* highlighted new layers in the regulation of seed dormancy and germination, including hormone transport in seeds and the epigenetic control of germination [10,14,16]. The knowledge of temporal and tissue-specific regulation of hormone metabolism and signaling during rice seed germination is still lacking. Epigenetic mechanisms can modulate the expression of genes related to germination. More details about chromatin modification, small RNA regulation, or post-translational modifications of hormone-related genes in rice germination are needed.

The main objective of seed dormancy or germination research is to guide rice production and new cultivar breeding in agricultural practice (i.e., we need to breed cultivars with an excellent germination ability to promote direct seeding practices). We can also develop effective seed treatment approaches to benefit seed germination and seedling emergence under adverse environments. Indeed, a high germination ability or strong seed vigor has not been selected as an important breeding trait because of the transplanting program in traditional rice production. Usually, the hybrid rice, including *japonica* and *indica* hybrid rice types, possesses stronger seed vigor during the germination stage than the inbred rice under various conditions. The loci or genes for seed germination in hybrid rice represent valuable resources for molecular breeding, and the mechanisms of seed germination enhancement need to be explored. The identification and application of the elite alleles of germination-controlling genes in agricultural conditions will still be challenging for a long time in the future.
